# Supplementary material for: Insertional oncogenesis by HPV70 revealed by multiple genomic analyses in a clinically HPV‐negative cervical cancer
Source: Genes Chromosomes Cancer. 2019 Sep 4;59(2):84–95. doi: 10.1002/gcc.22799 (PMC6916423; doi:10.1002/gcc.22799)
Supplement: Supplementary file 1 — Supplementary Figure 1 Sequence of the HPV70 reference genome is presented. *'s indicate the nucleotide was present in the viral DNA insertion in the tumor, and that the nucleotide was identical. Positions where the tumor nucleotides differed are highlighted in yellow and the change is shown. The gray highlighted segment is the URR between the L1 and E6 ORFs. [file GCC-59-84-s001.doc]

Supplementary Figure 1. Sequence of the HPV70 reference genome is presented. *‘s indicate the nucleotide was present in the viral DNA insertion in the tumor, and that the nucleotide was identical. Positions where the tumor nucleotides differed are highlighted in yellow and the change is shown. The gray highlighted segment is the URR between the L1 and E6 ORFs.

1 cttataacattttacaatcataatttaaaaaaagggaggcaccgaaaacggtcacgacc

***********************************************************

60 gaaaacggtgtatataaaaccatgcaaaagttgcttgcccatacggaatggcgcgattt

***********************************************************

119 cccaatcctgcagaacggccatacaaattgcctgacctgtgcacggcgctggacactac

***********************************************************

178 attgcacgacattacaatagactgtgtctattgtaaaacacagctacagcaaacagagg

***********************************************************

237 tatatgaatttgcatttagtgatttatttatagtatatagaaacggggagccatatgct

**************************************************A********

295 gcatgccaaaaatgtattaaatttcatgctaaagtaagggaactacggcattattcgaa

*************************T*******************************G*

355 ctcggtgtatgcaacaactttggaaagcataactaataccaagttatataatttatcaa

**************************************************G********

413 taaggtgcatgagttgcctgaaaccattgtgtccagcagaaaaattaaggcatgttaat

***********************************************************

472 accaaaagaagatttcaccaaatagcaggaagctatacaggacagtgccgacactgctg

******************AG***************************************

531 gaccagcaaccgggaggaccgcagacgtatacgaagagaaacacaagtataaatataaa

***********************************************************

591 tatgcatggaccacggccgacattgcaagagattgttttagatttatatccatacaatg

**********************G*******T****************************

650 aaatacagccggtcgaccttgtatgtcacgagcaattagaagattcagacaatgaaaca

*********************************************************G*

709 gatgaacccgaccatgtagttaatcaccaacaacaactactagccagacgggaagaacc

***********************************************************

768 acagcgtcacaaaatacagtgtatgtgttgtaagtgtaatactacactgcacttagtag

***********************************************************

827 tagaagcctcacaagagaacctgcgatctctactgcagctgtttatggagacactgtca

*************G*********************************************

886 tttgtgtgtccctggtgtgcatcgggaacccagtaacctgcaatggccaattgtgaagg

***********************************************************

945 tacagatggggatgggtcgggatgtaacggatggttcctagtacaggcaatagtagata

***********************************************************

1004 aacaaacgggcgacactgtgtcagaggacgaggacgaaaatgcaacagatacaggttca

*****************************T*****************************

1063 gacttggcagactttattgatgatactacagatatttgtgtacaggcagagcgcgagac

***********************************************************

1122 agcacaggtactgtataatatgcaagaggcccaaagggatgcacaatcagtgcgtgcct

***********************************************************

1181 taaaacgaaagtatggagggagcaatctaaataaaagtccttgtgcaaaaccgccaggc

***********************************************************

1240 gtacatagggaacaaagggtaacactacaagagctcccggtaaacatatgcaataaaca

***********************************************************

1299 ggcaagaacaaacgtgtattcagtaccagacagcggctatggcaatatggaagtggaaa

***********************************************************

1358 cagctgaagtggaggtaactgtagtaaataatacaaatggggaagaggaaggggaaaat

***********************************************************

1417 ggcggggaaaatggcggcagcatacgggaggagtgcagtagtgtagacagtgctattga

*****************************************************C*****

1476 tagtgagaatcaagatccacagtcacctactgcacagctaaaaacagtattacaggcta

************************************************G**********

1535 ataaccaaaaagccatactactatcacaatttaaacacacatatggattagcatttaac

*****A*G***************************************************

1594 gacctggtacgtacatttaaaagtgataaaaccatatgtactgactgggtagcagcaat

********************************T**************************

1653 atgtggagtaaatcccaccatagcagaaggctttaaaacactaattcagccatatgcgt

*************************C*********************************

1712 tatatacacatatacagtgtttggataccaaatatggagtgtatatactactattaatt

***********************************************T*****C*****

1771 agatataaatgtggaaaaaacaggataacagtaggcaaaggattaagtaaattattaca

***********************A***********************************

1830 tgtgccagaaagttgtatgctaattgaaccacctaaattgcgtagccctgttgcagcac

***********************************************************

1889 tgtattggtatagaactggaatgtctaatataagtgaagtgtcaggtactacgccagaa

***********************************************************

1948 tggatacagcgattaacagtaatacagcatggaatagatgacagtgtatttgacctgtc

***********************************************************

2007 tgatatggtacaatgggcatttgataatgatgtaacagaagacagtgacatagcatatg

***********************************************************

2066 gatatgcattattagcagatagtaatagtaatgctgcagcatttttaaaaagtaactgc

***********************************************************

2125 caggcaaaatatgtacgcgactgtgctacaatgtgcagacattataaaagggcacaaaa

***********************************************************

2184 aaaacaaatgactatggcgcaatggattaggtttagatgtgataaatgtgacgatgggg

G**********************************************************

2243 gcgactggcgaccaatagtgcaatttctaaggtatcaaggggtagaatttataaccttt

**************************T*G**A********************T******

2302 ttgtgtgcatttaaggagtttttaaagggcaccccaaagaaaaattgcatagtaataca

*****************************T*****************************

2361 gggaccaccaaacacaggcaagtcatacttttgtatgagtttaatgcactttttacaag

********************

2420 gtacagtaatttcatatgtaaattccactagtcatttttggttagagccacttgcagat

2479 gcaaaggtagcaatgttggatgatgccacaggcacatgctggtcatatttcgatacgta

2538 tatgagaaatgcattagatggaaatcctataagccttgacagaaaacatagacatttaa

2597 tacaaattaagtgtccacccatattaataacatccaataccaatcctgtagaggaaaat

2656 aggtggccatacctaactagcagactaacagtgtttacatttcctaatgcattcccatt

2715 tgaccaaaacaggaatccagtgtacacaatcaataataaaaactggaaaagttttttcc

2774 aaaagacttggtgcaaattagacttgcagcaggacgaggatgaaggagacaatgatgga

2833 aacactatcccaacgtttaaatgcgttacaggagaaaatactagaacattatgaacagg

2892 acagtaaactaatatatgatcaaatcaattattggaaatatgtgcgactggaaaatgca

2951 atattttatgcagcacgggaacgtggcatgcatactatagaccaccaggtggtgccacc

3010 aggcactacttcaaaagcaaaagcatatcaagctattgaactgcagatggccctagaga

3069 gccttgcacaaactgactttaataaagaggagtggacattaaaggacacaagtaatgaa

3128 atgtggcagacaaagccaaaacaatgttttaaaaaaaaaggtgttacagtggaggtgtg

3187 gtacgatggaaacaaggacaattctatgcattatgtagtgtggggagcaatatattata

3246 aaacacatacagacacgtggtgtaaaacagaagggtatgtggattactggggtatatat

3305 tatgtgcacgagcagcataagacatattatgaagtgtttaagcaggatgcacaaatgta

3364 tgggactagcggaaaatgggaagtgcattgtaatggcaacataattcattgtcctgact

3423 ctatgtacagtaccagtgacgacacagtacccactactgagcttactgcagaactacaa

3482 cacaccaccccggcccataccgccgcaacaaccccatgcaccaaaaaaactaagtcggc

3541 gccgtcttgcaagtgtggagtctccagaccctcagaaacagacggagtgttcgtggacc

3600 ttgttacaagtaaaggctgcaacaaacgacggcaccagtgttgtggtgacactacacct

3659 atagtgcatttaaaaggtgacaaaaatggtttaaagtgtcttaggtatcgattgcgaaa

3718 atttaattcattgtatgaaaatatttcatgtacttggcattggatagggggcaagggaa

3777 gtaaacatacaggtatactaactgtaacatatactactgaagcacaacgccaaaaattt

3836 ttggaaactgttagaattccacctagtgtacatgtatctgtgggatatatgacattgta

3895 acagcacatgctgtatgtatattgtatacatatcaatgattgcattggtgtttttggtg

3954 tggtttgctgtatgcttatatatatgttgcagtgtcccgcttttgccgtctgtgcattt

4013 gtgtgcgtatatgtggctacttttatttgtgtttattgttgtacataccacaccattgc

4072 aaatgttttgtatatatttactattttttatattgcctatgtggtttttacacatcctt

4131 tcagtatatgcttaagttgtgttgctgcatagtgtattgtacattacttgtttttacat

4190 ttatattgtaccaataaacatggtttctagccgtgcgtccaggcgtaagcgtgcatctg

4249 caacagacatatataaaacctgcaagcaatcaggcacatgtccgcctgatgttgttaat

4308 aaggtggagggtaccacactggctgataggtttttacaatgggctagtttaggtatttt

4367 tttgggtggtttgggaatcggtacgggtactggtactgggggccgcacagggtacattc

4426 ctttggggggtaggcctagtacagttgtagatgttacccctgcacgtcctcctgtggtt

4485 atagaacctgtaggacctacagaaccttctattgttcagttggtagaggaatctagtgt

4544 tgtttcctctggtacacccatccctacttttacaggcacatctgggtttgaaattacat

4603 cttctgcaaccacaacacctgctgtattagatattacccctgcttctgggtctgttcaa

4662 attagtaccactagttataccaatcctgcatttgctgatccatcgttaattgaggttcc

4721 acaaacaggtgaggtgtcaggcaatatatttgttactactccaacatctggaacacatg

4780 gatatgaagaaattcctatgcaggtttttgcctcacatggaacaggcacagaacctatt

4839 agtagtactcctgttcctggtgttagtcgtgtggcaggcccacgtttatatagtagggc

4898 ctatcatcaggttcgtgttaataattttgattttgtaacccgcccttcatcttttgtaa

4957 catttgacaatccagcttttgagcctggtgatacatccttaacatttgaacctgctgac

5016 acagctcctgatccagattttctggacattgttcgtttacatcggcctgctttaacctc

5075 acgacgcggaacagtacgctttagtaggcttggtaaaaaggccacaatgtttacccggc

5134 ggggtacacaaattggggcacaggttcattattatcatgatattagtaacattactgca

5193 acagaagacattgagatgcaacctttacttacctctgaatctacagatggtttatatga

5252 tatatatgcagatgcagatatagataatgcaatgttacatactacttctcatacaggtt

5311 ctacaggacctaggtcccatctttcatttccttctataccttctacagtgtctacaaaa

5370 tatagtaatacaaccattccatttactacttcttgggacatacctgtaaccactggccc

5429 tgacatagttttacctactgcatcccccaatttgccctttgtccctcctacatctatag

5488 ataccacagttgcaatagccattcagggctccaattattatttattgcctttattatat

5547 tattttctaaagaaacgtaaacgtattccctatttttttacagatggctttgtggcggt

5606 ctagtgacaacacggtgtatttgccacccccttctgtggcgaaggttgtcaatacagat

5665 gattatgtaacacgtacaggcatatattattatgctggaagctctcgcttattaacagt

5724 agggcatccttattttaaggtacctgtaaatggtggccgcaagcaggaaatacctaagg

5783 tgtctgcatatcagtatagggtatttagggtatccctacctgatcctaataagtttggc

5842 cttccggatccttccctttataatcctgacacacaacgcctggtatgggcctgtatagg

5901 tgtggaaattggtagaggccagccattgggcgttggcgttagtggacatcctttatata

5960 atagattggatgatactgaaaattctcatttttcctctgctgttagtacacaggacagt

6019 agggacaatgtgtctgtggactataagcaaacacagttatgtattataggctgtgttcc

6078 tgctatgggagagcactgggctaagggcaaggcctgtaagtccactcaacagggcgatt

6137 gtccaccattagaattagttaatactgcaattgaggatggcgatatgatagatacaggc

6196 tatggtgccatggactttcgtacattgcaggaaaccaaaagtgaggtaccactagatat

6255 ttgccaatccgtgtgtaaatatcctgattatttgcagatgtctgctgatgtatatgggg

*****************

6314 acagtatgtttttttgtttgcgcaaggaacagttgtttgccaggcacttttggaataga

**********************************A************************

6373 ggtggcatggtgggcgacacaataccttcagagttatatattaaaggcacggatatacg

***********************************************************

6432 tgagcgtcctggtactcatgtatattccccttccccaagtggctctatggtctcttctg

***T***********************************************T*******

6491 attcccagttgtttaataagccctattggttgcataaggcccagggacacaataatggc

***********************************************************

6550 atttgttggcataaccagttgtttattactgtggtggacactacacgtagtactaattt

***********************************************************

6609 tacattgtctgcctgcaccgaaacggccatacctgctgtatatagccctacaaagttta

******************************************************A****

6668 aggaatatactaggcatgtggaggaatatgatttacaatttatatttcaattgtgtact

***********************************************************

6727 atcacattaactgctgacgttatggcctacatccatactatgaatcctgcaattttgga

**************A********************************************

6786 caattggaatataggagttacccctccaccatctgcaagcttggtggacacgtataggt

***************C*******************************************

6845 atttacaatcagcagctatagcatgtcaaaaggatgctcctacacctgaaaaaaaggat

****************************G************G*****************

6904 ccctatgacgatttaaaattttggaatgttgatttaaaggaaaagtttagtacagaact

***********************************************************

6963 agatcagtttcctttggggcgcaaatttttactacaggtaggggctcgcagacgtccta

***********************************************************

7022 ctataggccctcgcaaacgccctgcgtcagctaaatcgtcttcctcagcctctaaacac

*************************A*********************************

7081 aaacggaaacgtgtgtccaagtaatgtatgtatgttgtatgctgtgtattattgtacta

**************************************************TA*******

7140 ttacatatttgtgtttttatgttgtatgcttgcacactgtttacatatttgtgtttgta

**********C*****C******************************************

7199 tgttgtatgcttgcacactgtactgtatatgtttgtcctggtacatatttgtggttgta

************************************************C**********

7258 tgtgtatatgttgcgtgctatgtgtatgttttagaagtatgtgtgtatgtatgtttttg

******************************G****************************

7317 ttaataaagtatgtatggaggtttcatttgtggttgcaccctgtgactaaggtgttgtc

*******************A***************************************

7376 cctgttttacatataataggagtgtgattaccaacatttcctacataattttatgccct

***********************************************************

7435 accctaaggtgtgtgtataccatttgtagtttatacatttatattttatagtgggttac

***********************CCT*********************************

7494 ctgtatacagcaacggccattttgtgtgaaaccgttttcggttgcatttggctttgtac

T*T*************************C******************************

7553 catcagttacccttataaaccttttgtatcagcaaaaacatgtcctgtaacctaagttc

********************G**********************T***************

7612 acctacatacttggcactactaacagttttagtggcgcacctacacttagtcatcatcc

******************************T****************************

7671 tgtccaggtgcactacaacaatgctttggcaaccttatgcacctccaccctgtctaata

*****************************************************A*****

7730 aagtgcttttaggcatgtattttacctgtttttacttacctaagagcatagttggcctg

******************T************************T***************

7789 tataacagcttttacatccaagaatgtgtcgtttggtgcaagttatattttgtgactaa

***********************************************************

7848 tatttttacagacctgtgtgcaaccgaaataggttgggcagacattcctatactttta

***************A******************************************
