# Supplementary material for: Insertional oncogenesis by HPV70 revealed by multiple genomic analyses in a clinically HPV‐negative cervical cancer
Source: Genes Chromosomes Cancer. 2019 Sep 4;59(2):84–95. doi: 10.1002/gcc.22799 (PMC6916423; doi:10.1002/gcc.22799)
Supplement: Supplementary file 2 — Supplementary Figure 2 Five of the split reads crossing the HPV70 E1^E4 5′ss to BCL11B exon 4 splice junction. HPV70 sequences are highlighted in blue. Human genome BCL11B sequences are highlighted in green. [file GCC-59-84-s002.doc]

Supplementary Figure 2.

Five of the split reads crossing the HPV70 E1^E4 5’ss to *BCL11B* exon 4 splice junction. HPV70 sequences are highlighted in blue. Human genome *BCL11B* sequences are highlighted in green.

**Split Read #1**

HPV70: 824-943 +strand

TAGTAGAAGCCTCACAGGAGAACCTGCGATCTCTACTGCAGCTGTTTATGGAGACACTGTCATTTGTGTGTCCCTGGTGTGCATCGGGAACCCAGTAACCTGCAATGGCCAATTGTGAAGGTAAAGATGAGCCTTCCAGCTACATTTGCA

Chr 14: 99,642,532-99,642,503 –strand

**Split Read #2**

HPV70: 809-843 +strand

CTACACTGCACTTAGTAGTAGAAGCCTCACAGGAGAACCTGCGATCTCTACTGCAGCTGTTTATGGAGACACTGTCATTTGTGTGTCCCTGGTGTGCATCGGGAACCCAGTAACCTGCAATGGCCAATTGTGAAGGTAAAGATGAGCCTT

Chr 14: 99,642,532-99,642,518 –strand

**Split Read #3**

HPV 70: 884-943 +strand

CATTTGTGTGTCCCTGGTGTGCATCGGGAACCCAGTAACCTGCAATGGCCAATTGTGAAGGTAAAGATGAGCCTTCCAGCTACATTTGCACAACATGCAAGCAGCCCTTCAACAGCGCGTGGTTCCTGCTGCAGCACGCGCAGAACACGC

Chr14: 99,642,532-99,642,443 -strand

**Split Read #4**

HPV70: 893- 943 +strand

GTCCCTGGTGTGCATCGGGAACCCAGTAACCTGCAATGGCCAATTGTGAAGGTAAAGATGAGCCTTCCAGCTACATTTGCACAACATGCAAGCAGCCCTTCAACAGCGCGTGGTTCCTGCTGCAGCACGCGCAGAACACGCACGGCTTCC

Chr 14: 99,642,532-99,642,434 -strand

**Split Read #5**

Chr14: 99,642,454-99,642,532 +strand

GCGTGCTGCAGCAGGAACCACGCGCTGTTGAAGGGCTGCTTGCATGTTGTGCAAATGTAGCTGGAAGGCTCATCTTTACCTTCACAATTGGCCATTGCAGGTTACTGGGTTCCCGATGCACACCAGGGACACACAAATGACAGTGTCTCC

HPV70: 943-873 -strand
