# Supplementary material for: Insertional oncogenesis by HPV70 revealed by multiple genomic analyses in a clinically HPV‐negative cervical cancer
Source: Genes Chromosomes Cancer. 2019 Sep 4;59(2):84–95. doi: 10.1002/gcc.22799 (PMC6916423; doi:10.1002/gcc.22799)
Supplement: Supplementary file 3 — Supplementary Figure 3 DNA sequence of BCL11B exon 3B (upper case letters, hg19 position chr14:99665867 to 99 665 710) with immediately flanking intron sequences (lower case letters). Upstream intron sequences include 3′ss consensus sequence (bold), t‐rich segment, and potential lariat site (ac). Downstream intron sequences include 5′ss consensus (bold). [file GCC-59-84-s003.doc]

Supplementary Figure 3.

Supplementary Figure 3A.

DNA sequence of *BCL11B* exon 3B (upper case letters, hg19 position chr14:99665867 to 99665710) with immediately flanking intron sequences (lower case letters). Upstream intron sequences include 3’ss consensus sequence (bold), t-rich segment, and potential lariat site (ac). Downstream intron sequences include 5’ss consensus (bold).

accacttttgattgtgtgattttgttt**cag**TCGAGAGCGCTCCAATCAGTCCCTCAGAAACAAGAAGGGGGAAAGAACCCTCTCACCAAATATCTGGGCGTCTGTGCAGATTCTCAGCCATTGAGAATATTGACAGCAATCAGAATTCCTCGGCTGTTGAAACTTCGCCTCTAATTGAACTGGGAAAG**gta**aacccagattcagggctctgcagggct

Supplementary Figure 3B.

The four RNAseq split reads crossing the HPV70 E1^E4 5’ss to *BCL11B* exon 3B splice junction. HPV70 sequences are highlighted in blue. Human genome *BCL11B* sequences are highlighted in green.

**Split read #1**

HPV70: 853-943 +strand

TCTCTACTGCAGCTGTTTATGGAGACACTGTCATTTGTGTGTCCCTGGTGTGCATCGGGAACCCAGTAACCTGCAATGGCCAATTGTGAAGTCGAGAGCGCTCCAATCAGTCCCTCAGAAACAAGAAGGGGGAAAGAACCCTCTCACCAA

Chr 14: 99,665,867-99,665,809 -strand

**Split read #2**

HPV70: 855-943 +strand

TCTACTGCAGCTGTTTATGGAGACACTGTCATTTGTGTGTCCCTGGTGTGCATCGGGAACCCAGTAACCTGCAATGGCCAATTGTGAAG

Chr 14: 99,665,867-99,665,807 –strand

**Split Read #3**

Chr14: 99,665,762-99,665,867 +strand

CTGTCAATATTCTCAATGGCTGAGAATCTGCACAGACGCCCAGATATTTGGTGAGAGGGTTCTTTCCCCCTTCTTGTTTCTGAGGGACTGATTGGAGCGCTCTCGACTTCACAATTGGCCATTGCAGGTTACTGGGTTCCCGATGCACAC

HPV70: 943-900 -strand

**Split Read #4**

Chr14: 99,665,731-99,665,867 +strand

CGAAGTTTCAACAGCCGAGGAATTCTGATTGCTGTCAATATTCTCAATGGCTGAGAATCTGCACAGACGCCCAGATATTTGGTGAGAGGGTTCTTTCCCCCTTCTTGTTTCTGAGGGACTGATTGGAGCGCTCTCGACTTCACAATTGGC

HPV70: 943-931 -strand

Supplementary Figure 3C.

Split reads crossing the *BCL11B* exon 3B to exon 4 splice junction. Exon 4 sequences are highlighted in green.

**Split Read #1**

chr 14: 99,665,779-99,665,710 -strand

CATTGAGAATATTGACAGCAATCAGAATTCCTCGGCTGTTGAAACTTCGCCTCTAATTGAACTGGGAAAGGTAAAGATGAGCCTTCCAGCTACATTTGCACAACATGCAAGCAGCCCTTCAACAGCGCGTGGTTCCTGCTGCAGCACGCG

chr 14: 99,642,532-99,642,453 -strand

**Split Read #2**

chr 14: 99,665,758-99,665,710 -strand

TCAGAATTCCTCGGCTGTTGAAACTTCGCCTCTAATTGAACTGGGAAAGGTAAAGATGAGCCTTCCAGCTACATTTGCACAACATGCAAGCAGCCCTTCAACAGCGCGTGGTTCCTGCTGCAGCACGCGCAGAACACGCACGGCTTCCGC

chr 14: 99,642,532-99,642,432 -strand

**Split Read #3**

chr 14: 99,665,725-99,665,710 -strand

AATTGAACTGGGAAAGGTAAAGATGAGCCTTCCAGCTACATTTGCACAACATGCAAGCAGCCCTTCAACAGCGCGTGGTTCCTGCTGCAGCACGCGCAGAACACGCACGGCTTCCGCATCTACCTGGAGCCCGGGCCGGCCAGCAGCTCG

chr 14: 99,642,532-99,642,399 –strand

**Split Read #4**

chr 14: 99,665,811-99,665,710 -strand

CAAATATCTGGGCGTCTGTGCAGATTCTCAGCCATTGAGAATATTGACAGCAATCAGAATTCCTCGGCTGTTGAAACTTCGCCTCTAATTGAACTGGGAAAGGTAAAGATGAGCCTTCCAGCTACATTTGCACAACATGCAAGCAGCCCT

chr 14: 99,642,532-99,642,485 -strand

**Split Read #5**

chr 14: 99,665,758-99,665,710 -strand

TCAGAATTCCTCGGCTGTTGAAACTTCGCCTCTAATTGAACTGGGAAAGGTAAAGATGAGCCTTCCAGCTACATTTGCACAACATGCAAGCAGCCCTTCAACAGCGCGTGGTTCCTGCTGCAGCACGCGCAGAACACGCACGGCTTCCGC

chr 14: 99,642,532-99,642,432 -strand

This split read was identical to Split Read #2. However the read pair partners for the them were different, thus confirming their independent origins.
