# Supplementary material for: Insertional oncogenesis by HPV70 revealed by multiple genomic analyses in a clinically HPV‐negative cervical cancer
Source: Genes Chromosomes Cancer. 2019 Sep 4;59(2):84–95. doi: 10.1002/gcc.22799 (PMC6916423; doi:10.1002/gcc.22799)
Supplement: Supplementary file 4 — Supplementary Figure 4 Sequences of RT‐PCR products shown in Figure 4B highlighting the HPV70 (blue), BCL11B exon 3B (gray), and BCL11B exon 4 (green) sequences of the PCR products generated from tumor RNA. [file GCC-59-84-s004.doc]

Supplementary Figure 4.

Supplementary Figure 4A.

Sequences of RT-PCR products shown in Figure 4B highlighting the HPV70 (blue), *BCL11B* exon 3B (gray), and *BCL11B* exon 4 (green) sequences of the PCR products generated from tumor RNA.

Main (lower) band; E1^exon4:

HPV70: 792-943 (+) strand

ATACTACACTGCACTTAGTAGTAGAAGCCTCACAGGAGAACCTGCGATCTCTACTGCAGCTGTTTATGGAGACACTGTCATTTGTGTGTCCCTGGTGTGCATCGGGAACCCAGTAACCTGCAATGGCCAATTGTGAAGGTAAAGATGAGCCTTCCAGCTACATTTGCA

Chr14: 99,642,532-99,642,502 (-) strand

Faint (upper) band; E1^exon 3B^exon 4:

HPV70: 806-943 (+) strand

ATACTACACTGCACTTAGTAGTAGAAGCCTCACAGGAGAACCTGCGATCTCTACTGCAGCTGTTTATGGAGACACTGTCATTTGTGTGTCCCTGGTGTGCATCGGGAACCCAGTAACCTGCAATGGCCAATTGTGAAGTCGAGAGCGCTCCAATCAGTCCCTCAGAAACAAGAAGGGGGAAAGAACCCTCTCACCAAATATCTGGGCGTCTGTGCAGATTCTCAGCCATTGAGAATATTGACAGCAATCAGAATTCCTCGGCTGTTGAAACTTCGCCTCTAATTGAACTGGGAAAGGTAAAGATGAGCCTTCCAGCTACATTTGCA

*BCL11B*:

exon 3B: Chr14: 99,665,867-99,665,710

exon 4: Chr14: 99,642,532-99,642,502

Supplementary Figure 4B.

Sequences of PCR products of the PCR shown in Figure 4C highlighting the HPV70 (blue), *BCL11B* (green), and micro-homology overlap (red) sequences.

Tumor:

AAAAACATACTGTCCCCATATACATCAGCACACACGTGTGTGCATACACACAAATACGCGTGTATGTACAAATACATATGCACATATGCTCTCACATCTACACATATACACATGCATGCACATATATACACTTAGTCATGTACACACATGCACACCCCCACATTAACACAC

CIN3:

AAAAACATACTGTCCCCATATACATCAGCACACACGTGTGTGCATACACACAAATACGCGTGTATGTACAAATACATATGCACATATGCTCTCACATCTACACATATACACATGCATGCACATATATACACTTAGTCATGTACACACATGCACACCCCCACATTCACACACAC
